# Protocol for a systematic review evaluating psychometric properties and gender-related measurement (non)invariance of self-report assessment tools for autism in adults

**DOI:** 10.1186/s13643-024-02604-2

**Published:** 2024-07-19

**Authors:** Nora Uglik-Marucha, David Mason, Hannah Belcher, Francesca Happé, Silia Vitoratou

**Affiliations:** 1 Psychometrics and Measurement Lab, Biostatistics and Health Informatics Department, Institute of Psychiatry, Psychology and Neuroscience, King’s College, London, SE5 8AF UK; 2 Social, Genetic and Developmental Psychiatry Centre, Institute of Psychiatry, Psychology and Neuroscience, King’s College, London, SE5 8AF UK; 3 Health Service and Population Research, Institute of Psychiatry, Psychology and Neuroscience, King’s College, London, SE5 8AF UK

**Keywords:** Autism, Adults, Self-report, Measures, COSMIN, Measurement invariance, Gender bias, Psychometric properties

## Abstract

**Background:**

Given the recent evidence on gender differences in the presentation of autism, there is an increasing concern that current tools for autism do not adequately capture traits more often found in women. If tools for autism measure autistic traits differently based on gender alone, their validity may be compromised as they may not be measuring the same construct across genders. Measurement invariance investigations of autism measures can help assess the validity of autism constructs for different genders. The aim of this systematic review is to identify and critically appraise the psychometric properties of all self-report tools for autism in adults that meet two criteria: (a) they have been published since or included in the NICE (2014) recommendations, and (b) they have undergone gender-related measurement invariance investigations as part of their validation process.

**Methods:**

A search of electronic databases will be conducted from 2014 until the present using MEDLINE, Embase, and PsycINFO using predefined search terms to identify eligible studies. The search for grey literature will include sources such as OpenGrey, APA PsycEXTRA, and Scopus. Two reviewers will independently screen titles, abstracts, and full texts for eligibility. The references of included studies will be searched for additional records. The methodological quality of the studies will be evaluated using the COSMIN Risk of Bias checklist, while psychometric quality of findings will be assessed based on criteria for good measurement properties and ConPsy checklist. The quality of the total body of evidence will be appraised using the approach outlined in the modified GRADE guidelines.

**Discussion:**

This systematic review will be among the first to assess the psychometric properties and gender-related measurement invariance of self-reported measures for autism in adults that were published since (or included in) NICE (2014) guidelines. The review will provide recommendations for the most suitable tool to assess for autism without gender bias. If no such measure is found, it will identify existing tools with promising psychometric properties that require further testing, or suggest developing a new measure.

**Systematic review registration:**

The protocol has been registered at the International Prospective Register of Systematic Reviews (PROSPERO). The registration number is CRD42023429350.

**Supplementary Information:**

The online version contains supplementary material available at 10.1186/s13643-024-02604-2.

## Background

The diagnosis of autism spectrum disorder, referred to hereafter as autism,^1^ is based on the presence of behavioural features related to social interaction and communication differences, and restricted interests, and repetitive behaviours (RRBs) (which includes sensory differences). These traits can vary based on several clinical specifiers—language skills, intellectual abilities, co-occurring conditions, genetic, or environmental factors, and support needs—and their various combinations, thus contributing to the complexity and variation in autism presentation. This variability, denoted by the term ‘spectrum’ in the DSM-5, is further illuminated by genetic findings [2] and research on brain structure [3]. As there are no reliable biomarkers for autism [4], screening and diagnosis rely on behavioural observation, developmental history, and self-report questionnaires, presenting challenges due to the diverse and complex nature of the condition.

Traditionally, autism has been predominantly associated with males, as diagnostic criteria were primarily derived from observations of male children [5–7]. Research indicates a higher prevalence of autism in males across age groups [8, 9], with recent estimates revealing a gender disparity in diagnoses without intellectual disability, around 4:1 in clinical samples [10, 11] and 3:1 in population-based samples [12]. This ratio discrepancy suggests that a portion of autistic females with high autistic traits may meet clinical criteria but do not receive a formal diagnosis [12]. Indeed, a predictive model relying on population-based data has estimated that around 39% more girls should be diagnosed as autistic, potentially indicating a ‘leaky pipeline’ in the assessment of autism, particularly at the screening level [13]. However, it is essential to recognize the increasing number of diagnoses in females in recent years, even though they continue to be diagnosed at older ages compared to males across almost every age group [14].

There has been a concern in the literature that this high male to female ratio may be due to existing screening and diagnostic tools operating differently based on the gender of the respondent and thus lead to different clinical decisions due to gender alone. The concern that existing questionnaires do not fully capture the presentations of autistic traits in women has been raised particularly in light of the use of predominantly male samples in the validation of autism assessment tools [6, 15, 16]. This suggests that the measures may have been developed around the traits of autistic males, potentially making them less sensitive to traits more commonly reported in autistic women [17]. This is particularly important given that recent evidence suggests there are gender differences in the presentation of autism [17, 18] and that a ‘female’ presentation of autism may exist [19, 20].

When it comes to gender/sex^2^ (hereafter to be referred to as ‘gender’) differences in scores on autism instruments, findings are mixed, depending on how autism domains are assessed—either at a broad (encompassing the two main domains of autism, namely social communication and interaction, and RRBs) or narrow (focusing on subdomains, which include specific behavioural exemplars within each main domain) construct level—and the specific measures used. A recent systematic review and meta-analysis by Cruz et al. [21] revealed that autistic males tend to score higher on the Autism Diagnostic Observation Schedule (ADOS) [22] for total levels of autistic traits compared to autistic females, with no gender differences observed on other evaluated measures. Specifically focusing on the broad construct of social interactions, autistic males showed higher scores on the ADOS, indicating more challenges than autistic females; however, the opposite trend was found for parent/caregiver/teacher-report measures [21]. No gender differences in scores were found on instruments assessing communication and RRBs at the broader construct level [21]. Large-scale studies and reviews focusing on gender differences in RRBs suggest that autistic males tend to exhibit more RRBs than autistic females. This trend holds true at both broader [23–27] and narrower [18] construct levels of RRBs, with the latter specifically observed only for stereotyped behaviours and restricted interests subdomains. When examining gender differences in social communication, broad construct level meta-analyses often find no gender differences [27–29], while narrow-level ones do [17]. However, it remains unclear whether these score discrepancies accurately reflect gender differences in autistic traits or if traits more commonly observed in autistic women are not adequately captured by existing instruments [17, 18, 21].

### The importance of measurement invariant tools in the assessment of autism

If the existing assessment tools for autism measure autistic traits differently depending on the gender of a respondent alone, their validity to assess for autism can be compromised, that is, they may not measure the same construct in different genders. The gender bias of the measure can affect the number of people who are identified or flagged during the screening procedure [30]. This can lead to not being referred for diagnosis or affect the eligibility for interventions or receiving support. In research, the use of biased tools can lead to invalid conclusions with regard to comparisons between groups or the effectiveness of trials [31, 32].

To ensure that inferences are a genuine reflection of differences in the underlying construct rather than of the bias of the measurement tool, it is crucial that the construct is measured equivalently across different groups [32], which in psychometric literature is referred to as measurement invariance (equivalence or comparability) or non-differential item functioning. A measurement tool is said to be biased (measurement non-invariant or to exhibit differential item functioning, DIF) if some of its items do not assess the underlying construct equivalently across different groups of individuals [33] or conditions (multiple time points or raters). For instance, if men tend to have higher odds to endorse autism questionnaire items (traits, behaviours) than women even when their given level of autism is the same, this would indicate gender-related measurement non-invariance of the tool, that is measurement bias due to gender. When a measurement is biased, then the scores do not reflect the quantity that we seek to measure alone, but also the group effect. That being the case, the groups cannot be compared (as one will be either overestimated or underestimated), and any cut-off points would need to be group-specific. It is important to note that measurement invariance should not be conflated with the equality of *scores* across groups or conditions, wherein both groups exhibit identical scores on the underlying construct. Rather, measurement invariance ensures that the scores can be compared in a fair and meaningful manner, much as how a ruler is expected to measure the height of individuals of different genders in the same way without overestimating or underestimating it based solely on their gender membership [34–36]. Therefore, establishing measurement invariance does not imply that scores are equal across different groups (they may or may not be), but instead ensures that if there are any observed differences across groups, they are not due to methodological bias but rather reflect genuine differences in the trait.

This highlights the critical role of testing measurement invariance during the process of scale validation, especially for the use in clinically heterogeneous conditions such as autism, and given the recent evidence on gender differences in the condition. However, at the same time, it is important to acknowledge that our understanding of measurement invariance and its significance is still evolving, with varying perspectives within the field regarding its relevance in comparative research [37–41]. Consequently, any assertions regarding measurement invariance should be interpreted within this context in mind.

### Measurement invariance from the methodological standpoint

In a recent review, Leitgöb et al. [42] identified four generations of methodological approaches for testing measurement invariance. In this study, we will focus on the most popularly used methods in the literature within the first generation of approaches, which test for exact (full or partial) invariance. These are typically assessed using confirmatory factor analysis (CFA) methods within structural equation modelling (SEM) framework, such as multiple group CFA [43] or the multiple indicators multiple causes model (MIMIC) [44]. In the item response theory (IRT) framework, measurement non-invariance is often referred to as DIF, and it has a conceptual overlap with CFA methods. For a comprehensive overview of recent advancements in statistical methods for testing measurement invariance, we recommend referring to the reviews conducted by Leitgöb et al. [42] and Somaraju et al. [45].

In the SEM framework, four main types of measurement invariance exist, described for instance in Widaman and Reise [46]: configural, metric (weak), scalar (strong), and residual (strict) invariance. These four types involve a series of hierarchical factor models, for which each subsequent model implements more parameter (loadings, intercepts, error variances) constraints to those in the previous model. At each successive step, the models are compared through the series of nested model tests to evaluate them for measurement invariance. The first level of hierarchy is an assessment of the equivalence of measurement construct (the configural invariance), which is a baseline model that allows for the parameters to be freely estimated across groups or conditions. This step assesses whether the number of latent constructs and the specific items loading onto them are the same across groups. Each further step adds parameter constraints across groups starting with the loadings constraints to test for equivalence of factor loadings (metric invariance), that is, to assess if each item contributes to the latent construct to a similar degree across groups. In the third step, intercepts or thresholds are held invariant (scalar invariance) to test if items have the same expected response across groups for the same absolute trait levels. The final step involves adding constraints to error variances (strict invariance) to assess if items are explained by the trait equivalently, and it is not always possible to assess (for instance, in categorical or mixed items). Moreover, the invariance of item residuals has no bearing on the interpretation of latent mean differences, and therefore is not a prerequisite for testing mean differences [47]. Establishing configural, metric, and scalar invariance of the tool is thus sufficient for meaningful group or condition comparisons on their observed or latent scores for the construct (that is, score differences or structural invariance).

If full measurement invariance is not supported, the models can be tested for partial (metric, scalar, and strict) invariance, that is, to allow for some violations of measurement invariance by freely estimating a subset of parameters across groups or conditions. Items with released item parameter constraints indicate DIF for those items, and only partial invariance may hold for the rest of the items. However, the degree to which partial measurement invariance is acceptable is still a matter of debate [48] and is discussed on a case-by-case level. Additionally, in recent years, various effect size measures have been proposed by researchers (see for instance Groskurth et al. [49], Gunn et al. [50]; Meade [51], Nye & Drasgow [52], and Nye et al. [53]) to assess the degree of non-invariant parameters and to evaluate the practical significance of detected non-invariance. However, clear benchmarks for these measures have yet to be established.

Within the framework of IRT, DIF is employed to examine measurement non-invariance of individual items in a scale. In a manner akin to SEM, comparison tests are conducted between nested models. The key parameters in IRT are the difficulty (*b*) and the discrimination (*a*) parameters, with DIF arising when these parameters differ across groups or conditions. When DIF is present for the discrimination parameter (that is, an item appears more strongly related to the trait for one group or condition), it indicates the presence of non-uniform DIF [54]. Conversely, uniform DIF [54] pertains to difficulty parameter and occurs when an item is more severe for one group. Non-uniform DIF is conceptually similar to metric non-invariance in SEM, while uniform DIF is somewhat analogous to scalar non-invariance [55].

### Measurement invariance investigations of autism assessments

There is a scarcity of strong evidence to support gender-related measurement invariance in the self-report measurement tools for autism in adults. Two investigations into measurement invariance of Autism Quotient-10 (AQ-10) [56], which is a recommended screening measure by the National Institute for Health and Care Excellence (NICE) [57], by Murray et al. [58, 59] employed item response theory framework to evaluate items’ gender (non) bias. The initial study revealed two items in the AQ-10 that were biased, with one item biased against females and the other item against males. In the replication study, it was found that males had a higher probability of endorsing one item of AQ-10 than women for the same level of trait. However, this specific item was not biased in the initial study, and the items that were previously identified as biased no longer were gender non-invariant in the replication study. In both studies, no substantial bias was found at the test score level. Recent measurement invariance investigation [60] using 7076 responses of general population on Autism Quotient [61] under eleven model frameworks revealed that with respect to AQ-10 specifically, eight items were found to be gender-biased, where four were more likely to be endorsed by men and the remaining four by women. Considering all eleven models of AQ together, only two items were not biased. For the remaining items, the probability of endorsement was influenced by gender alone. Despite the results on measurement invariance tests being inconsistent on the degree of gender bias of AQ-10 items, it is still widely used to screen for autism, thus posing a risk of missing proportion of individuals that should be referred for a diagnosis.

The current review aims to identify further gender^3^-related measurement invariance investigations in the literature and evaluate whether current psychometric tools for autism in adults are gender (non)biased. Specifically, the systematic review will appraise the methodological and psychometric quality of all studies that validate self-report assessment tools for autism in adults and have conducted measurement invariance investigations with respect to gender as a part of the tool validation process. The tools to be included in the review will involve those that were published since (and included in) the update on evidence for the assessment of autism in adults outlined in NICE [57] recommendations. The update corresponds to the changes in Diagnostic and Statistical Manual of Mental Disorders-5 (DSM-5) [62] for autism, whereby it combined closely related diagnoses, such as Asperger’s syndrome or pervasive developmental disorder-not otherwise specified (PDD-NOS), under a single label of autism spectrum disorder. Unlike the previous edition, DSM-5 permits autistic individuals to be diagnosed with other conditions in addition to autism. The updated diagnostic criteria also combined differences in social interactions and communication into a single criterium, thereby reducing the three categories outlined in the DSM-4 to two. Therefore, limiting the search to measures published (and included) since NICE [57] guidelines will ensure they will reflect current diagnostic criteria.

Although there have been three systematic reviews to assess psychometric properties of screening and diagnostic measures for autism in adults [63–65], they did not investigate the gender-related measurement invariance of these tools. Furthermore, two of them [27, 29] examined only limited number of psychometric properties of the included tools, whereas the current review aims to assess each measure on several measurement properties outlined in the COnsensus-based Standards for the selection of health status Measurement INstruments (COSMIN) [66, 67] Risk of Bias checklist and the Contemporary Psychometrics checklist (ConPsy) [68]. Therefore, it is necessary to conduct a more comprehensive review that evaluates the psychometric properties and gender-related measurement (non)invariance of self-report measures for autism in adults. Ultimately, this review will offer evidence-based knowledge that can inform the selection of the most suitable measure for assessing autism in adults without gender bias. It is of urgent significance to provide clinicians, researchers, and service providers about synthesized evidence on psychometric properties and gender-related measurement (non)invariance of the existing tools to enable valid assessments of autism traits in both men and women or inform about the need to develop tools that will measure autism more equitably in both genders.

The proposed systematic review aims to address the following questions:

1. Which self-report autism assessment measures for adults (published since or included in NICE 2014 recommendations) are, and which are not, gender-biased, as indicated by measurement invariance investigations?
2. What is the methodological and psychometric quality of these measures?
3. What self-report measures are the most suitable for assessing autism in adults without gender bias?

## Methods

The systematic review will be conducted in adherence to the following protocol, and any changes that will occur throughout the study’s duration will be reported. This protocol follows the COSMIN guidelines for systematic reviews of Patient-Reported Outcome Measures [69] and the Preferred Reporting Items for Systematic Review and Meta-Analysis Protocols (PRISMA) guidelines (please see Additional file 1 for PRISMA-P checklist) [70]. The protocol has been registered on the International Prospective Register of Systematic Reviews (PROSPERO) database (registration number: CRD42023429350).

### Search strategy

#### Published literature

A systematic search of electronic databases will be conducted to identify relevant published literature using the Medical Literature Analysis Retrieval System Online (MEDLINE), Excerpta Medica database (Embase), and Psychological Information Database (PsycINFO) via Ovid interface. A reference list of included papers and existing systematic reviews on the psychometric properties of screening and diagnostic measures for autism in adults [27–29] will be searched for additional records. If new articles emerge upon examining a reference list of included studies, the search strategy will be adjusted to be broader, and the search repeated. Experts in the field of autism will be contacted about potential forthcoming publications on psychometric measures. Measures included in the NICE guidelines will be searched by using an online search engine.

Key words relating to construct of interest (here, autism), the population of interest (adults), the type of instrument (self-report measures), and psychometric properties (validity, measurement invariance) will be used to identify relevant literature for this review. Medical Subject Headings (MeSH) and free-text terms pertaining to key concepts will be combined using Boolean operators as follows: autism AND adults AND measures AND psychometric properties. Similar terms will be combined into sets using OR operator, for instance with respect to autism: autis* OR asperger* OR pervasive developmental*. Please see Table 1 for key words and their synonyms. The search strategy was developed in MEDLINE and then adjusted for each database. The collection of search terms was consulted with the experts on psychometrics (SV) and autism (FH, HB, DM) (please see Table 2 in the Appendix for a detailed search strategy of the key terms for each database).

**Table 1:** Key words and their synonyms

Key concepts	Synonyms
Autism	autis*, asperger*, pervasive developmental*
Adults	adult*
Measures	measure*, scale*, questionnaire*, inventory*, checklist*, schedule*, instrument*, assessment*, survey*, tool*, outcome*, screen*, self-report*, diagnos*
Psychometric properties	psychometric*, measurement*, valid*, measurement invarian*, measurement equivalen*, differential item*, dif

The databases will be searched from 2014, which corresponds to the year the latest update to the NICE guidelines was published, until the present. The searches will be limited to studies available in English language.

#### Grey literature

To search through the grey literature, the System for Information on Grey Literature in Europe (OpenGrey), APA PsycEXTRA, and Scopus will be used, along with searching the websites of major publishers (Pearson and Western Psychological Services) to identify any potential instruments not previously found. Conference proceedings will not be included in the review due to their limited information and potential differences in data presentation compared to full study reports.

### Eligibility criteria

The inclusion of studies will follow the outlined criteria:

#### Construct of interest

Eligible articles are those that validate self-report assessment tools for autism in adults, which have conducted measurement invariance investigations with respect to gender as a part of the tool validation process. Instruments that only assess for one of the two core autism domains (either only differences in communication and social interaction or presence of restricted, repetitive behaviours and interests) will be excluded.

#### Population of interest

Studies that validate the measures in adult population aged 18 years and over will be eligible for inclusion. If studies include participants over 16 years old, the study sample should have at least ≥ 50% of people aged 18 and over for it to be included in the review.

#### Measures

Eligible assessment tools are those that were developed using a reflective model, which assumes that all items within the measure are manifestations of a common construct and are correlated (as opposed to the formative model). Specifically, these instruments will be eligible for inclusion if they rely on an individual’s own report of autistic traits. Thus, studies validating parent- or teacher-report measures, or tools that rely on the assessment of observed behaviour by an examiner will be excluded. Articles that only use the self-report tool as an outcome measurement instrument will be excluded due to demonstrating only indirect evidence on the psychometric properties of the tool (for instance, in randomized clinical trials) [30].

#### Psychometric properties

Studies that evaluate psychometric properties of self-report measures for autism in adults are eligible for inclusion if they report at least on measurement invariance with respect to gender out of ten psychometric properties outlined by the COSMIN taxonomy (please see *Methodological quality* section for further details).

#### Study design

Quantitative and mixed-methods studies that aim to validate a measurement tool for autism in adults will be eligible for inclusion.

#### Setting

No restrictions will be applied to the type of setting.

#### Additional limitations

Only full-text articles will be included in the review as the abstracts usually offer incomplete information on the psychometric properties of the scales, which would hinder the assessment of psychometric properties of instruments and quality of the studies. If the full-text article cannot be accessed, the authors of the study will be contacted for a copy. Studies that developed and validated instruments exclusively in English will be eligible for inclusion in order to eliminate the need to adjust for potential translation or cultural effects in the measurements.

### Selection of articles

The resulting articles from the database searches will be imported to Endnote 20 and screened independently by NUM and DM to detect eligible studies based on their titles and abstracts. The full-text reports of potentially relevant articles will be independently assessed by the two reviewers against the full inclusion criteria outlined above. If an article is considered eligible by at least one reviewer, the discrepancies will be discussed. In case the consensus is not achieved, SV will be consulted to resolve it. A reference list of included papers and unpublished literature will be searched for additional records by NUM, and the articles considered eligible will be re-checked for their inclusion by DM. To evaluate the agreement between the reviewers, inter-rater reliability test will be implemented to warrant the consistency at the title and abstract screening, full-text screening, the data extraction, and quality assessment stages. The inter-rater agreement will be calculated using Cohen’s kappa (κ) [71], whereby the values of 0.81–1 are indicative of very good agreement, 0.61–0.80 are considered good, 0.41–0.60 suggest moderate agreement, 0.21–0.40 are considered fair, and < 0.20 are indicative of poor agreement. The steps involved in the selection of articles will be displayed in a PRISMA flow chart.

### Evaluation of methodological and psychometric quality

The review will assess the included studies on their methodological quality and the psychometric quality of the measurement tool, which will be assessed independently by the two reviewers. Any disagreements between the reviewers will be discussed to reach unanimity, and the third reviewer will be consulted if this cannot be achieved. Agreement between the raters will be evaluated using Cohen’s κ.

#### Methodological quality

The methodological quality of studies that evaluate the measurement properties of instruments for autism in adults will be assessed using COSMIN Risk of Bias checklist for systematic reviews of Patient-Reported Outcome Measures [66, 67]. The COSMIN checklist is a standardized tool for assessing study quality of psychometric studies on each of ten measurement properties, namely PROM development (Box 1; *content validity*), content validity (Box 2; *content validity*), structural validity (Box 3; *internal structure*), internal consistency (Box 4; *internal structure*), cross-cultural validity/measurement invariance (Box 5; *internal structure*), reliability (Box 6; *remaining measurement properties*), measurement error (Box 7; *remaining measurement properties*), criterion validity (Box 8; *remaining measurement properties*), hypotheses testing for construct validity (Box 9; *remaining measurement properties*), and responsiveness (Box 10; *remaining measurement properties*) (please see Table 3 in the Appendix for definitions of each measurement property). For each study, only the boxes corresponding to measurement properties that were evaluated in the article will be completed as usually only a limited number of psychometric properties are assessed per study. For instance, if a study assessed internal consistency and measurement invariance of a measure, only two boxes (Box 4 and 5) will be completed.

Each measurement property is rated either as ‘very good’ (4), ‘adequate’ (3), ‘doubtful’ (2), or ‘inadequate’ (1). According to COSMIN, the overall score for each study is determined by using the worst rating of any standard in the box. However, the quality rating based on the ‘worst score counts’ principle can hinder the identification of subtle differences in methodological quality between studies [72]. Thus, the quality rating for each measurement property will be given through a ratio between the total score minus the minimum possible score and the maximum possible score minus the minimum possible score, which will be then multiplied by 100 to produce a percentage score [73]. The rating for each property will be assigned as either inadequate if the percentage score is between 0 and 25%, doubtful (25.1 to 50%), adequate (50.1 to 75%), or very good (75.1 to 100%).

#### Psychometric quality

The evaluation of psychometric properties of the measurement tools will involve a three-stage process, wherein (1) findings from each study will be evaluated and graded, (2) the findings from all studies per measure will be summarized, and (3) the quality of evidence pertaining to the psychometric properties will be graded.

Findings from individual studies will be rated per each measurement property based on criteria for good measurement properties (please see Table 4 in the Appendix) [74]. These will be rated as either sufficient ( +), insufficient ( −), or indeterminate (?). Subsequently, all findings on each measurement property per measurement tool will be qualitatively summarized into an overall rating (sufficient ( +), insufficient ( −), inconsistent ( ±), or indeterminate (?)). However, a 75% rule will be employed, that is, the measurement property will receive an overall rating of either sufficient ( +) or insufficient ( −) if ≥ 75% of the studies reporting on that property for a specific instrument will be rated sufficient ( +) or insufficient ( −), respectively. Otherwise, the overall rating for that property will be inconsistent ( ±). The measurement property for a specific scale will receive an overall rating of indeterminate (?) if all studies are indeterminate (?). The overall ratings will be then used to grade the quality of evidence as either high, moderate, low, or very low quality using the approach outlined in modified version of Grading of Recommendations, Assessment, Development and Evaluations (GRADE; modified for grading the quality of the evidence in systematic reviews for PROMs) [69]. These ratings will be presented per measurement property.

To complement the recommendations outlined in the criteria for good measurement properties (Table 4 in the Appendix) [74], the ConPsy [68] checklist will be employed to evaluate the accuracy of the analyses used to validate the instruments in addition to their resulting indices. The checklist is presently being updated by the original author (SV) and NUM to incorporate the assessment of measurement invariance and accommodate the latest developments in the field of psychometrics. The structured checklist will include the evaluation of reliability (internal consistency, test–retest, inter-rater), validity (content, construct, and criterion validity), dimensionality (structural validity), and measurement invariance. The instruments will be evaluated in two ways: (1) rating will be provided based on whether suitable statistical methods were used and (2) a score will be assigned based on the resulting indices of the analyses. The quality scores for each psychometric property will be derived from published criteria and summed to provide a global quality score.

### Data extraction

The data extraction of included articles will be performed independently by NUM and DM to reduce errors and bias. The disagreements will be resolved through discussions and if necessary, with SV acting as the third reviewer to achieve the consensus. Inter-rater reliability will be calculated for the agreement assessment between the reviewers. Where necessary, the authors of articles will be contacted to resolve uncertainties.

#### Measures

For each measure identified through the search strategy, the extracted data table was designed based on COSMIN guidelines to include information on authorship, year of publication, country, study title, instrument name (abbreviation), objectives, number of items, number of factors, response options, range of scores, assessed psychometric properties, population, sample size, and its characteristics (please see Table 5 in the Appendix for the data extraction form).

#### Psychometric properties

The results of measurement properties that will be extracted will correspond to eight (COSMIN Boxes 3 to 10; Boxes 1 and 2 are not included as they evaluate content validity) out of ten measurement properties evaluated by COSMIN needed to employ the checklist. The data extraction table for measurement properties results [75] for each study is available in Table 6 in the Appendix, which will be used to enter the ratings for both methodological and psychometric qualities. Overall rating for a measure per property and graded quality of evidence will be entered into a summary of findings [75] table to be found in Table 7 in the Appendix. Results pertaining to measurement invariance investigations with respect to gender will be extracted to a table designed for this study (Table 8 in the Appendix), which includes the name of the instrument (abbreviation), framework for testing measurement invariance, number of items, number of biased items per gender, number of non-biased items, and type of invariance.

### Data synthesis

The general characteristics of the included studies will be summarized and presented. Descriptive statistics on the number of measures, evaluated psychometric properties, and number of gender (non)biased items will be computed. The overall rating will be provided for each study per each measurement property for both the methodological quality and the psychometric quality of the measure. The summary of findings table (Table 7 in the Appendix) will be used to offer recommendations for the most appropriate self-report measure to assess autism in adults without gender bias. When identifying the most appropriate instrument, measurement invariance with respect to gender will be deemed the most crucial measurement property. The assessment tools will be categorized into three categories outlined by COSMIN [66, 67]: (A) self-report measures that have potential to be recommended as the most suitable measure for the construct and population of interest; (B) self-report measures that may have the potential to be recommended, but further validation studies are needed (scales not categorized in A or C); and (C) self-report measures that should not be recommended. A rationale for assigning instruments into one of the three categories will be provided, along with guidance for further validation of the measures (if applicable).

## Discussion

To the best of the authors’ knowledge, this will be the first systematic review to appraise the psychometric properties and gender-related measurement invariance of self-reported measures for autism in adults that were published since (or included) in NICE [57] guidelines and provide recommendations for the most suitable tool to assess for autism without gender bias. Evidence of gender-related measurement invariance of scales reinforces the validity of the autistic traits they measure as equally valid for measuring autism for men and women. In psychological research, it is a crucial prerequisite for valid testing of construct differences across groups and enables the researchers to distinguish test bias from the true difference in the construct. In clinical decision-making, measurement invariance ensures that gender alone does not bias the scores on screening tools and allows for fair referral for diagnostic assessments and receiving appropriate support.

If no gender-related measurement invariant measures are found, this review will also identify whether any of the existing measures have promising psychometric properties for which further amendments and psychometric testing are necessary or if new measures need to be developed. When scales with favourable measurement properties are available, emphasis should be placed on further evaluating these measures rather than creating new ones.

The protocol for systematic review presented in this article is subject to certain limitations. Firstly, only measurement tools validated in English and studies published in English will be eligible for inclusion. Thus, results pertaining to gender-related measurement invariance and remaining measurement properties published in languages other than English will not be used in the synthesis of evidence. Secondly, interpretability and feasibility will not be evaluated as a part of the systematic review because they are not regarded as psychometric properties according to the COSMIN taxonomy used in this review, despite having been shown to be important in evaluating the overall quality of a measurement tool [75].

### Supplementary Information

Additional file 1. PRISMA-P 2015 Checklist.docx

## Data Availability

Not applicable.

## Appendix

**Table 2:** Detailed search strategy pertaining to the key terms and number of results (as of 24/05/2023) for each database. The searches will be limited to studies published from 2014 to present (May 2023) and available in English language

**Database** (results)	**Concept 1**	**Concept 2**	**Concept 3**	**Concept 4**
	*Autism*	*Measures*	*Psychometric properties*	*Adulthood*
MedLINE (1050)	1. exp Autistic Disorder/ 2. exp Asperger Syndrome/ 3. exp Autism Spectrum Disorder/ 4. autis*.mp 5. asperger*.mp 6. pervasive developmental*.mp 7. 1 or 2 or 3 or 4 or 5 or 6	8. exp “Surveys and Questionnaires”/ 9. exp Self Report/ 10. measure*.mp 11. scale*.mp 12. questionnaire*.mp 13. inventory*.mp 14. exp Checklist/ 15. checklist*.mp 16. schedule*.mp 17. instrument*.mp 18. assessment*.mp 19. survey*.mp 20. tool*.mp 21. exp Patient Reported Outcome Measures/ 22. outcome*.mp 23. screen*.mp 24. diagnos*.mp 25. self-report*.mp 26. 8 or 9 or 10 or 11 or 12 or 13 or 14 or 15 or 16 or 17 or 18 or 19 or 20 or 21 or 22 or 23 or 24 or 25	26. exp Psychometrics/ 27. psychometric*.mp 28. measurement*.mp 29. valid*.mp 30. measurement invarian*.mp 31. measurement equivalen*.mp 32. differential item*.mp 33. dif.mp 34. 26 or 27 or 28 or 29 or 30 or 31 or 32 or 33	35. exp Adult/ 36. adult*.mp 37. 35 or 36
Embase (3199)	1. exp autism/ 2. exp Asperger syndrome/ 3. autis*.mp 4. asperger*.mp 5. exp “pervasive developmental disorder not otherwise specified”/ 6. pervasive developmental*.mp 7. 1 or 2 or 3 or 4 or 5 or 6	8. exp questionnaire/ 9. exp self report/ 10. measure*.mp 11. scale*.mp 12. questionnaire*.mp 13. inventory*.mp 14. exp checklist/ 15. checklist*.mp 16. schedule*.mp 17. instrument*.mp 18. assessment*.mp 19. exp autism assessment/ 20. exp clinical assessment/ 21. exp clinical assessment tool/ 22. exp psychologic assessment/ 23. exp symptom assessment/ 24. exp patient assessment/ 25. survey*.mp 26. tool*.mp 27. exp patient-reported outcome/ 28. outcome*.mp 29. exp screening test/ 30. exp screening/ 31. screen*.mp 32. diagnos*.mp 33. self-report*.mp 34. 8 or 9 or 10 or 11 or 12 or 13 or 14 or 15 or 16 or 17 or 18 or 19 or 20 or 21 or 22 or 23 or 24 or 25 or 26 or 27 or 28 or 29 or 30 or 31 or 32 or 33	35. exp psychometry/ 36. psychometric*.mp 36. exp validity/ 37. measurement*.mp 38. valid*.mp 39. measurement invarian*.mp 40. measurement equivalen*.mp 41. differential item*.mp 42. dif.mp 43. 34 or 35 or 36 or 37 or 38 or 39 or 40 or 41 or 42	44. exp adult/ 45. adult*.mp 46. 44 or 45
PsycINFO (1075)	1. exp Autism Spectrum Disorders/ 2. exp Autistic Traits/ 3. autis*.mp 4. asperger*.mp 5. pervasive developmental*.mp 6. 1 or 2 or 3 or 4 or 5	7. exp Questionnaires/ 8. exp Surveys/ 9. exp Self-Report/ 10. exp Measurement/ 11. measure*.mp 12. questionnaire*.mp 13. scale*.mp 14. inventory*.mp 15. exp “Checklist (Testing)”/ 16. checklist*.mp 17. schedule*.mp 18. instrument*.mp 19. exp Psychological Assessment/ 20. assessment*.mp 21. exp “Mental Health and Illness Assessment”/ 22. survey*.mp 23. tool*.mp 24. exp Patient Reported Outcome Measures/ 25. outcome*.mp 26. exp Screening Tests/ 27. exp Screening/ 28. screen*.mp 29. diagnos*.mp 30. self-report*.mp 31. 7 or 8 or 9 or 10 or 11 or 12 or 13 or 14 or 15 or 16 or 17 or 18 or 19 or 20 or 21 or 22 or 23 or 24 or 25 or 26 or 27 or 28 or 29 or 30	32. exp Psychometrics/ 33. psychometric*.mp 34. measurement*.mp 35. exp Test Validity/ 36. exp Test Construction/ 37. valid*.mp 38. exp Measurement Invariance/ 39. exp Differential Item Functioning/ 40. Dif.mp 41. measurement equivalen*.mp 42. measurement invarian*.mp 43. differential item*.mp 44. 32 or 33 or 34 or 35 or 36 or 37 or 38 or 39 or 40 or 41 or 42 or 43	45. adult*.mp

**Table 3:** COSMIN definitions of domains, measurement properties, and aspects of measurement properties [66, 67]

Term			Definition
*Domain*	*Measurement property*	*Aspects of a measurement property*	
Reliability			The degree to which the measurement is free from measurement error
Reliability (extended definition)			The extent to which scores for patients who have not changed are the same for repeated measurement under several conditions: e.g. using different sets of items from the same PROM (internal consistency); over time (test‐retest); by different persons on the same occasion (inter-rater); or by the same persons (i.e. raters or responders) on different occasions (intra-rater)
	Internal consistency		The degree of the interrelatedness among the items
	Reliability		The proportion of the total variance in the measurements which is due to ‘true’^a^ differences between patients
	Measurement error		The systematic and random error of a patient’s score that is not attributed to true changes in the construct to be measured
Validity			The degree to which a PROM measures the construct(s) it purports to measure
	Content validity		The degree to which the content of a PROM is an adequate reflection of the construct to be measured
		Face validity	The degree to which (the items of) a PROM indeed looks as though they are an adequate reflection of the construct to be measured
	Construct validity		The degree to which the scores of a PROM are consistent with hypotheses (for instance with regard to internal relationships, relationships to scores of other instruments, or differences between relevant groups) based on the assumption that the PROM validly measures the construct to be measured
		Structural validity	The degree to which the scores of a PROM are an adequate reflection of the dimensionality of the construct to be measured
		Hypotheses testing	Item construct validity
		Cross-cultural validity	The degree to which the performance of the items on a translated or culturally adapted PROM are an adequate reflection of the performance of the items of the original version of the PROM
	Criterion validity		The degree to which the scores of a PROM are an adequate reflection of a ‘gold standard’
Responsiveness			
	Responsiveness		Item responsiveness
Interpretability^b^			Interpretability is the degree to which one can assign qualitative meaning ‐ that is, clinical or commonly understood connotations – to a PROM’s quantitative scores or change in scores

^a^The word ‘true’ must be seen in the context of the CTT, which states that any observation is composed of two components—a true score and error associated with the observation. ‘True’ is the average score that would be obtained if the scale were given an infinite number of times. It refers only to the consistency of the score, and not to its accuracy. ^b^Interpretability is not considered a measurement property, but an important characteristic of a measurement instrument

**Table 4:** Criteria for good measurement properties

Measurement property	Rating	Criteria
Structural validity	+	**CTT** CFA: CFI or TLI or comparable measure > 0.95 OR RMSEA < 0.06 OR SRMR < 0.08^a^ **IRT/Rasch** No violation of *unidimensionality*^b^: CFI or TLI or comparable measure > 0.95 OR RMSEA < 0.06 OR SRMR < 0.08 *AND* no violation of *local independence*: residual correlations among the items after controlling for the dominant factor < 0.20 OR Q3’s < 0.37 *AND* no violation of *monotonicity*: adequate looking graphs OR item scalability > 0.30 *AND* adequate *model fit* IRT: *χ*^2^ > 0.001 Rasch: infit and outfit mean squares ≥ 0.5 and ≤ 1.5 OR Z-standardized values > − 2 and < 2
	?	CTT: not all information for ‘ + ’ reported IRT/Rasch: model fit not reported
	-	Criteria for ‘ + ’ not met
Internal consistency	+	At least low evidence^c^ for sufficient structural validity AND Cronbach’s alpha(s) ≥ 0.70 for each unidimensional scale or subscal^e^
	?	Criteria for “At least low evidence^c^ for sufficient structural validity^d^” not met
	-	At least low evidence^c^ for sufficient structural validity^d^ AND Cronbach’s alpha(s) < 0.70 for each unidimensional scale or subscale^e^
Reliability	+	ICC or weighted Kappa ≥ 0.70
	?	ICC or weighted Kappa not reported
	-	ICC or weighted Kappa < 0.70
Measurement error	+	SDC or LoA < MIC^d^
	?	MIC not defined
	-	SDC or LoA > MIC^d^
Hypotheses testing for construct validity	+	The result is in accordance with the hypothesis^f^
	?	No hypothesis defined (by the review team)
	-	The result is not in accordance with the hypothesis^f^
Cross-cultural validity \ measurement invariance	+	No important differences found between group factors (such as age, gender, language) in multiple group factor analysis OR no important DIF for group factors (McFadden’s R^2^ < 0.02)
	?	No multiple group factor analysis OR DIF analysis performed
	-	Important differences between group factors OR DIF was found
Criterion validity	+	Correlation with gold standard ≥ 0.70 OR AUC ≥ 0.70
	?	Not all information for ‘ + ’ reported
	-	Correlation with gold standard < 0.70 OR AUC < 0.70
Responsiveness	+	The result is in accordance with the hypothesis^f^ OR AUC ≥ 0.70
	?	No hypothesis defined (by the review team)
	-	The result is not in accordance with the hypothesis^f^ OR AUC < 0.70

The criteria are based on, e.g. Terwee et al. [74] and Prinsen et al. [76]; *AUC*, area under the curve; *CFA*, confirmatory factor analysis; *CFI*, comparative fit index; *CTT*, classical test theory; *DIF*, differential item functioning; *ICC*, intraclass correlation coefficient; *IRT*, item response theory; *LoA*, limits of agreement; *MIC*, minimal important change; *RMSEA*, root mean square error of approximation; *SEM*, standard error of measurement; *SDC*, smallest detectable change; *SRMR*, standardized root mean residuals; *TLI*, Tucker–Lewis index; “ + ” = sufficient, “ − ” = insufficient, “?” = indeterminate; ^a^To rate the quality of the summary score, the factor structures should be equal across studies; ^b^Unidimensionality refers to a factor analysis per subscale, while structural validity refers to a factor analysis of a (multidimensional) Patient-Reported Outcome Measure; ^c^As defined by grading the evidence according to the GRADE approach ^d^This evidence may come from different studies; ^e^The criteria ‘Cronbach alpha < 0.95’ was deleted, as this is relevant in the development phase of a PROM and not when evaluating an existing PROM; ^f^The results of all studies should be taken together and it should then be decided if 75% of the results are in accordance with the hypotheses

**Table 5:** Data extraction form for measures

Authors	Year of publication	Country	Study title	Instrument (abbreviation)	Objectives	Number of items	Number of factors	Response options	Range of scores	Assessed psychometric properties	Population	Sample
										Content validity (Box 2) Structural validity (Box 3) Internal consistency (Box 4) Cross-cultural validity / measurement invariance (Box 5) Reliability (Box 6) Measurement error (Box 7) Criterion validity (Box 8) Hypotheses testing for construct validity (Box 9) Responsiveness (Box 10)	General Clinical	Size Gender Age Ethnicity

**Table 6:** Data extraction form for results of measurement properties from each study [75]

**Measure (ref)**	**Structural validity**			**Internal consistency**			**Cross-cultural validity / measurement invariance**			**Reliability**		
	*n*	*Meth quality*	*Result (rating)*	*n*	*Meth quality*	*Result (rating)*	*n*	*Meth quality*	*Result (rating)*	*n*	*Meth quality*	*Result (rating)*
*Measure 1 (ref)*												
*Measure 1 (ref)*												
**Pooled or summary result (overall rating)**												
**Measure(ref)**	**Measurement error**			**Criterion validity**			**Hypotheses testing**			**Responsiveness**		
	*n*	*Meth quality*	*Result (rating)*	*n*	*Meth quality*	*Result (rating)*	*n*	*Meth quality*	*Result (rating)*	*n*	*Meth quality*	*Result (rating)*
*Measure 1 (ref)*												
*Measure 1 (ref)*												
**Pooled or summary result (overall rating)**												

*Meth quality:* Methodological quality

**Table 7:** Summary of findings Table [75]

	Summary or pooled results	Overall rating	Quality of evidence
**Structural validity**			
* Measure 1*			
* Measure 2*			
**Internal consistency**			
* Measure 1*			
* Measure 2*			
**Cross-cultural validity / measurement invariance**			
* Measure 1*			
* Measure 2*			
**Reliability**			
* Measure 1*			
* Measure 2*			
**Measurement error**			
* Measure 1*			
* Measure 2*			
**Criterion validity**			
* Measure 1*			
* Measure 2*			
**Hypotheses testing**			
* Measure 1*			
* Measure 2*			
**Responsiveness**			
* Measure 1*			
* Measure 2*

**Table 8:** Data extraction table for results of measurement invariance investigations

**Measure (ref)**	**Framework for testing MIN**	**Number of items**	**Measurement noninvariance**			**Type of invariance**
			**Number and label of biased items**		**Number and label of non-biased items**	
			*Female*	*Male*		
*Measure 1 (ref)*						

*MIN:* Measurement invariance

## Abbreviations

AQ: Autism Quotient
AQ-10: Autism Quotient-10
CONPSY: Contemporary Psychometrics checklist
COSMIN: COnsensus-based Standards for the selection of health status Measurement Instruments
DIF: Differential item functioning
DSM-5: Diagnostic and Statistical Manual of Mental Disorders-5
Embase: Excerpta Medica database
GRADE: Grading of Recommendations, Assessment, Development and Evaluations
IRT: Item response theory
MEDLINE: Medical Literature Analysis Retrieval System Online
MeSH: Medical Subject Headings
NICE: National Institute for Health and Care Excellence
PDD-NOS: Pervasive developmental disorder-not otherwise specified
PRISMA: Preferred Reporting Items for Systematic Review and Meta-Analysis
PsycINFO: Psychological Information Database
SEM: Structural equation modelling

## Footnotes

1 ‘Autism’ is used instead of the synonymous medical DSM-5 term ‘autism spectrum disorder’ in line with the views of members of autism community to encompass that autistic experiences include strengths as well as difficulties [1].

2 Studies vary with regard to whether they record information about sex or gender, and frequently use these terms interchangeably.

3 While acknowledging the distinction between sex (biological characteristics) and gender (sociocultural attributes), this study will consider investigations into measurement invariance with respect to both sex and gender to reflect the interchangeable use of these terms in the literature.
